# Non-Invasive Tear Break-Up Detection with the Kowa DR-1α and Its Relationship to Dry Eye Clinical Severity

**DOI:** 10.3390/ijms232314774

**Published:** 2022-11-25

**Authors:** Stephen Pflugfelder, Lauren Nakhleh, Yasushi Kikukawa, Shin Tanaka, Takuya Kosugi

**Affiliations:** 1 Department of Ophthalmology, Baylor College of Medicine, Houston, TX 77030, USA; 2 Kowa Company, Ltd., Tokyo 103-8433, Japan; 3 Kowa Ophthalmic Research Laboratories, Kowa Research Institute Inc., Boston, NC 27560, USA

**Keywords:** break-up, dry, eye, interferometer, non-invasive, tear, stability

## Abstract

The purpose of this study is to compare visual versus software detection of non-invasive tear break-up with the KOWA DR-1α tear interferometer and investigate the relationship between non-invasive tear break-up time (NIBUT) and dry eye clinical severity. Tear interferometry with the KOWA DR-1α, together with a standardized comprehensive ocular surface/tear evaluation, was performed on 348 consecutive eyes. Investigator visually detected or software detected non-invasive tear break-up and NIBUT were measured and compared on a subset of these examinations. The relationship between software-detected NIBUT and categorical dry eye severity based on irritation symptoms and corneal and conjunctival dye staining scores was determined. The sensitivity of visual (frame-by-frame) or software detected non-invasive tear break-up in eyes with tear instability (FBUT < 10) was similar (range 63–69%). NIBUT, measured visually or by software, had a correlation coefficient of 0.87. NIBUT was significantly lower in severity levels 2 and 3 compared to levels 0 + 1, and level 3 was significantly lower than level 2. In conclusion, there is a good correlation between investigator visually detected and software-detected tear break-up and tear break-up time in the KOWA DR-1α interferometric fringe images. Software-detected NIBUT is a clinically relevant measure of dry eye clinical severity.

## 1. Introduction

Dry eye is a common eye condition with a reported prevalence ranging from 5 to 30% of the population depending on the diagnostic criteria [1,2,3,4,5]. Tear instability is a hallmark of dry eye and a key feature in consensus definitions proposed by the Dry Eye Workshop II (DEWS II), and by an international panel of clinicians who considered dry eye to be a disease characterized by a persistently unstable and/or deficient tear film [6,7]. 

Tear break-up can be evaluated clinically by visual detection of discontinuities in the tear film following fluorescein instillation or using non-invasive methods [8]. The KOWA DR-1α (Kowa Company, Ltd., Tokyo, Japan) aims to observe and record a video of the interference image of the tear film illuminated with white light [8]. Users can replay the recorded image on the instrument’s monitor to observe the status of the tear film layer.

**Figure 1:**
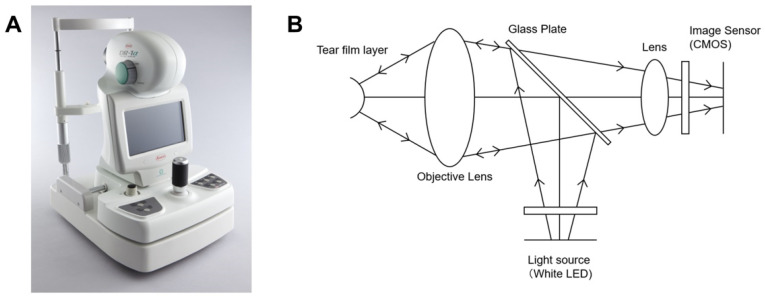
(**A**) The KOWA DR-1a instrument. (**B**) KOWA DR-1a optics. To view light reflected from the tear film layer, the KOWA DR-1α uses two parallel polarizers so that only the reflected image of the illumination light is captured by the camera, and the external light is not taken into the camera.

The surface of the tear film is covered by a thin lipid layer of approximately 100 nm thick. When the tear film surface is illuminated with white light, several interference patterns appear, resulting from the difference in two specular reflection light paths obtained between the front and back surfaces of the lipid layer. To view light reflected from the tear film layer, the KOWA DR-1α instrument uses two parallel polarizers so only the reflected image of the illumination light is captured by the camera, and the external light is not taken into the camera (Figure 1). As a result, background images, such as the iris and sclera, do not appear, and the interference image of the tear film layer can be efficiently observed.

**Figure 2:**
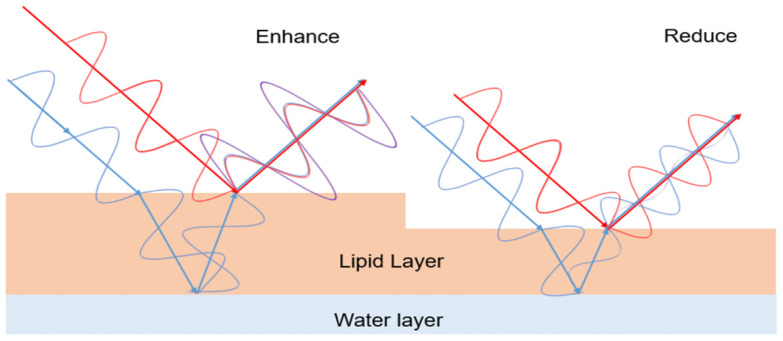
Interferometry is now an established technique for clinical evaluation of tear lipid layer kinetics and tear break-up. Left. The light intensity is enhanced when the phase of light reflected at the surface of the lipid layer matches the phase of light reflected at the interface between the lipid layer, of a certain thickness, and the water layer. Right. The light is reduced when the lipid layer is changed to a thickness such that the phase of the two reflected lights is shifted.

**Figure 3:**
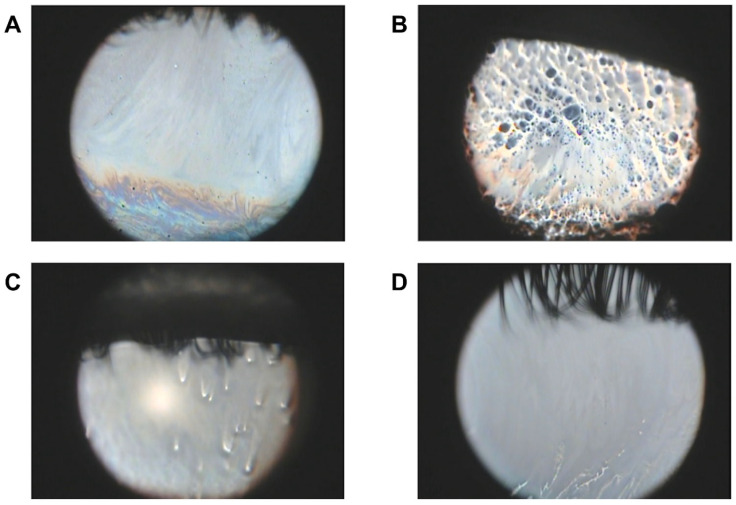
Four patterns of tear film break-up have been observed. (**A**) normal interference color images without break-up; (**B**) area break-up; (**C**) spot break-up; (**D**) line break-up.

The KOWA DR-1α interferometer was developed to evaluate the kinetics of the tear film lipid layer in both normal subjects and patients with dry eye disease. This instrument revealed that lipid layer kinetics are related to the tear film condition or blink pattern. Interferometry is now an established technique for clinical evaluation of tear lipid layer kinetics and tear break-up (Figure 2). Four patterns of tear film break-up have been observed (Figure 3). The software’s ability to accurately detect tear break-up would represent a great advance in clinical utilization of this technique because clinicians would not have to manually review exams and measure tear break-up.

The purpose of this study is to compare visual versus software detection of non-invasive tear break-up (NIBUT) with the KOWA DR-1α tear interferometer and the relationship between non-invasive tear break-up time (NIBUT) with dry eye clinical severity. There was concluded to be a good correlation between investigator visually detected and software-detected tear break-up and tear break-up time in the KOWA DR-1α interferometric fringe images. Software-detected NIBUT is a clinically relevant measure of dry eye clinical severity. 

## 2. Results

Table 1 shows the results for 179 patients (348 eyes). Patients were classified as aqueous tear deficiency (ATD), meibomian gland disease (MGD), normal, and others based on published criteria [9,10]. The clinical features of these patients are summarized in Table 1.

### 2.1. Detection of Non-Invasive Tear Break-Up

Analysis was performed on subgroup of eyes (32 eyes) in which the investigator used a frame button to advance the video frame-by-frame to identify the initial break more precisely. The sensitivity of non-invasive tear break-up detection in this group was 63% for visual grading, and 69% for software grading in eyes with FBUT < 10 s (Table 2).

### 2.2. Correlation between Software and Visually Detected NIBUT

**Figure 4:**
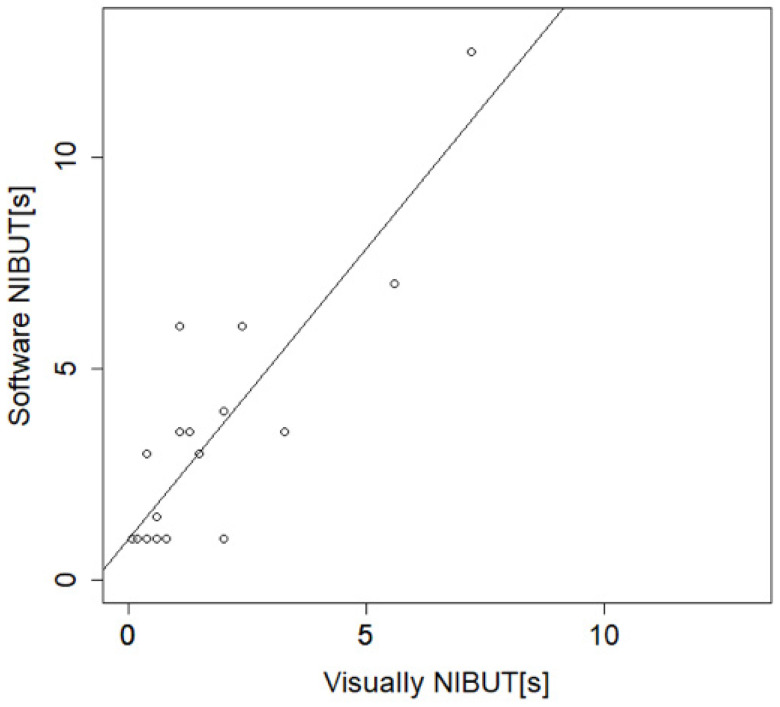
Graph showing correlation between visually detected, and software detected NIBUT in subset of patients where frame button was used by the investigator to identify the frame in which break-up was detected (*n* = 20; ID = 21–40). Correlation coefficient is 0.87. One exam (ID33R) was not evaluated because patient had only partial blinks. Subjects consisted of ATD (40%), MGD (50%) and normal (10%). Mean severity score for this group is 2.11 ± 0.81.

The correlation was calculated between software and visually detected NIBUT in exams reviewed with a frame button to precisely identify the frame in which break-up can be detected (Table 2). The correlation coefficient is 0.87 in this group (Figure 4). 

### 2.3. Relationship between NIBUT and Categorical Clinical Severity 

**Figure 5:**
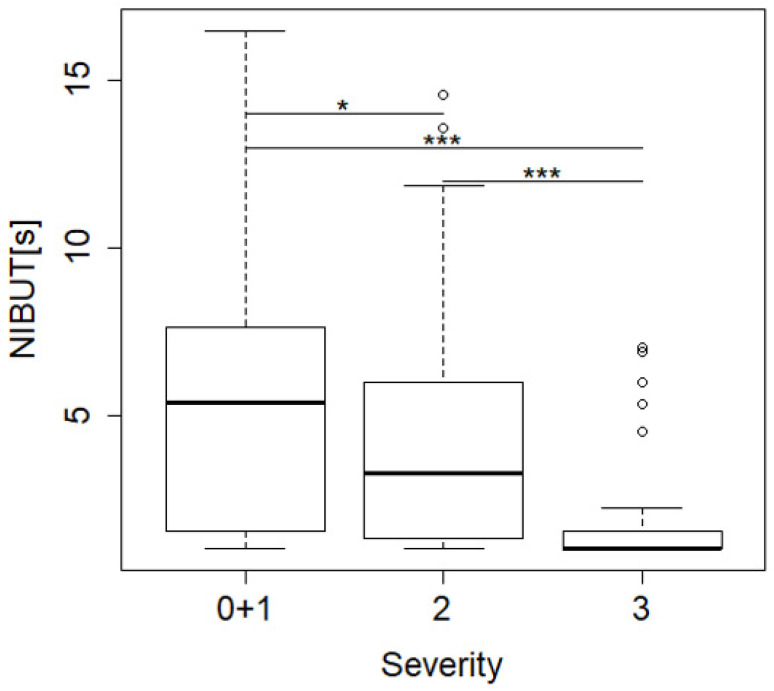
Association between software detected NIBUT with categorical clinical severity using DEWSII criteria. Levels 0 + 1 were combined. Steel–Dwass test *** *p* < 0.001, * *p* < 0.05.

The relationship between software-detected NIBUT and categorical severity of dry eye was evaluated in 270 eyes. As level 1 severity dry eye is often considered equivocal, the normal and level 1 groups were combined (0 + 1) for statistical comparison with more severe levels of dry eye. NIBUT was significantly lower in level 2 and level 3 compared to level 0 + 1 and level 3 was also significantly lower than level 2 (Figure 5).

## 3. Discussion

This study compared the sensitivity of non-invasive tear break-up detection in tear interferometric videos taken with the KOWA DR-1α, either by visual inspection or using software. Additionally, the correlation between NIBUT, measured visually or with software, and the relationship between NIBUT and categorical clinical severity, were determined. The sensitivity of the detection of tear break-up was similar between the methods. Similarly, there was high correlation between the two methods for measuring NIBUT. As further evidence of the clinical utility of this technology, software-detected NIBUT was found to correlate with clinical severity. Tear break-up was also found to be decreased in more severe dry eye by conventional FBUT [11].

These results suggest that software detection of tear break-up has similar sensitivity to clinician detection of NIBUT in tear interferometry videos. The utilization of software to detect abnormal tear break-up in a standardized fashion in exams performed by a technician would speed and simplify the work-up of tear film deficiency. Additional tear break-up parameters, including the location and pattern of break-up, could be analyzed in the interferometry videos. This type of analysis also provides a digital record for improved diagnostic classification, severity grading and determining the effects of dry eye therapies on tear film stability. 

In summary, software detection of NIBUT and other parameters of tear stability could represent a major advance in standardized evaluation of tear disorders and could improve the efficiency of clinical evaluation of these conditions. 

## 4. Materials and Methods

This study was approved by the Baylor College of Medicine Institutional Review Board (IRB; Protocol number H-51925), and all research adhered to the tenets of the Declaration of Helsinki. A retrospective chart review of all patients who had a comprehensive ocular surface examination for dry eye at the Alkek Eye Center from 2019 to 2022 was performed. Patients were excluded if they had conjunctivochalasis, keratoneuralgia or non-tear-film-related eye discomfort. 

All patients had standard panel of tear film and ocular surface tests, including Symptom Assessment in Dry Eye (SANDE) symptom questionnaire, interferometric analysis of tear stability with the KOWA DR-1α, optical coherence tomography measurement of tear meniscus height (Avanti, Optovue, CA, USA), biomicroscopic exam, fluorescein tear break-up time, cornea fluorescein staining and conjunctival lissamine green staining. These tests were performed by previously reported methods [9]. The severity of the ocular surface disease was graded 0–3 using previously reported severity criteria [10,12]. 

The instrument was calibrated daily using a standardized procedure. Light intensity was set at 10:00 and area of illumination was set to wide. The exam was performed for 30 s in each eye and the video was saved. 

In a subset of exams, NIBUT was measured by the investigator in one eye per subject by reviewing the video. The end of a blink and the first detection of tear break-up was marked on the video timeline using the software. The video segment containing tear break-up was re-reviewed on a frame-by-frame basis to accurately measure the tear break-up time.

For a software analysis of NIBUT, a previously constructed image classification model to detect characteristics of the KOWA DR-1α tear film image was developed as a method to detect tear break-up [13]. Analysis of the KOWA DR-1α videos using software implementing this detection method was used to detect inter blink intervals and tear break-up. NIBUT with software was measured as the time elapsed between the last blink and the detection of the first break-up. If patients can no longer refrain from blinking before the tear film break-up, the blink intervals were measured as the break-up time. 

Statistical tests were carried out using the statistical programming language R (version 3.6.1, The R Foundation for Statistical Computing, Vienna, Austria). The statistical significance was set at a *p* < 0.05 for all the analyses. The Steel–Dwass test was used to compare software detection of NIBUT between the severity groups. Sample size was calculated using the assumption of a ± 0.5 margin of error for severity and confidence interval of 95% (equation provided in Supplementary Information).

## Figures and Tables

**Table 1:** Clinical features.

Group	Age	Sex (F/M)	SANDE (R/L)	FBUT (R/L) s.	Cornea FL Staining (R/L)	Conjunctiva LG Staining (R/L)	Severity (0–3)
ATD *N* = 49; 95 eyes	60.39 ± 14.80	F:42 M:7	R: 4.04 ± 1.09 L: 4.10 ± 1.10	R: 4.37 ± 2.85 L: 3.94 ± 2.71	R: 3.57 ± 3.44 L: 4.00 ± 3.82	R: 2.41 ± 2.06 L: 2.78 ± 2.16	2.29 ± 0.68
MGD *N* = 67; 131 eyes	61.69 ± 15.31	F:51 M:16	R: 3.67 ± 1.27 L: 3.84 ± 1.32	R: 5.34 ± 2.84 L: 5.31 ± 2.97	R: 1.78 ± 3.02 L: 1.80 ± 3.19	R: 1.06 ± 1.47 L: 1.11 ± 1.29	1.72 ± 0.57
Normal *N* = 23; 44 eyes	48.43 ± 16.11	F:19 M:4	R: 2.00 ± 1.65 L: 2.33 ± 1.85	R: 8.43 ± 2.46 L: 8.90 ± 2.28	R: 0.17 ± 0.49 L: 0.14 ± 0.48	R: 0.13 ± 0.34 L: 0.33 ± 0.58	0
Others *N* = 40; 78 eyes	62.75 ± 12.02	F:28 M:12	R: 3.67 ± 1.38L: 3.74 ± 1.31	R: 6.72 ± 3.36 L: 6.84 ± 3.19	R: 0.90 ± 1.62L: 1.05 ± 1.79	R: 0.95 ± 1.62 L: 0.82 ± 1.45	1.18 ± 0.91

ATD = aqueous tear deficiency; MGD = meibomian gland disease; Others = pinguecula, keratoneuralgia, epithelial basement membrane disease, floppy eyelid; SANDE = symptom assessment questionnaire in dry eye; FBUT = fluorescein break-up time; FL = fluorescein, LG = lissamine green; severity score.

**Table 2:** Comparison of the sensitivity of NIBUT detection in a subset of subjects *. FBUT < 10 s (32 eyes).

		Software		
		Break-Up Detect	Break-Up Not-Detect	Total
**Investigator** ^**†**^	**Break-Up** **Detected**	19	1	20
	**Break-Up** **Not Detected**	3	9	12
	**Total**	22	10	32

Sensitivity: visual detection: 63% (20/32), software detection: 69% (22/32). * Tear break-up was identified in this subgroup using a frame button to advance the video frame-by-frame to identify up the initial break more precisely. Subjects consisted of ATD (48%), MGD (48%) and normal (2%). Mean severity score for these eyes is 1.81 ± 0.86. A sample size of 14 was calculated to show between group differences with a confidence interval of 95%. ^†^ Investigator SCP.

## Data Availability

All data is included in the manuscript.

## Supplementary Materials

The following supporting information can be downloaded at: https://www.mdpi.com/article/10.3390/ijms232314774/s1, Equation S1: Sample size calculation.
