# Supplementary material for: Non-Invasive Tear Break-Up Detection with the Kowa DR-1α and Its Relationship to Dry Eye Clinical Severity
Source: Int J Mol Sci. 2022 Nov 25;23(23):14774. doi: 10.3390/ijms232314774 (PMC9737539; doi:10.3390/ijms232314774)
Supplement: Supplementary file 1 [file ijms-23-14774-s001.zip › ijms-2020409-supplementary.pdf]

Supplementary information

Equation S1: Sample size calculation.

$$n \geq \frac{4z_{\alpha/2}^2 \sigma_0^2}{\delta^2}$$

n: sample size

(1-α) = 0.95 (95%) (Confidence interval) (α=0.05)

Z-score (α/2) =1.960

δ: margin of error (±δ/2)

σ: standard deviation
